# Public Awareness and Willingness to Vaccinate Against Herpes Zoster: A Nationwide Cross-Sectional Study in Poland

**DOI:** 10.3390/vaccines12121393

**Published:** 2024-12-11

**Authors:** Justyna Grudziąż-Sękowska, Kuba Sękowski, Zuzanna Grześczyk-Nojszewska, Agnieszka Kamińska, Radosław Sierpiński, Janusz Ostrowski, Jarosław Pinkas, Mateusz Jankowski

**Affiliations:** 1 School of Public Health, Centre of Postgraduate Medical Education, 01-826 Warsaw, Poland; 2 Department of Ophthalmology, Faculty of Medicine, Collegium Medicum, Cardinal Stefan Wyszynski University, 01-938 Warsaw, Poland; 3 Faculty of Medicine, Collegium Medicum, Cardinal Stefan Wyszynski University, 01-938 Warsaw, Poland

**Keywords:** herpes zoster, vaccination, public awareness, vaccine hesitancy, health communication, risk factors, comorbidities, adult vaccination, Poland

## Abstract

Objectives: Herpes zoster (HZ), caused by varicella zoster virus reactivation, affects a significant portion of the population, leading to substantial morbidity. Vaccination is highly effective in preventing HZ, yet awareness and uptake remain low. This study assessed awareness and willingness to vaccinate against HZ in Poland following the introduction of a reimbursement policy. Methods: A nationwide cross-sectional survey (September 2024) using a computer-assisted web interview (CAWI) method collected data from 1137 adults. Factors associated with HZ vaccine awareness and willingness were analyzed using logistic regression models. Results: Only 47% of respondents reported awareness of the HZ vaccine. Television was the primary information source (52%). Factors associated with awareness included chronic disease status (aOR = 1.35, 1.02–1.80, *p* = 0.04). Willingness to vaccinate was reported by 63.7% of eligible participants, with factors such as the absence of children (aOR = 1.30, 1.01–1.69, *p* = 0.04) and moderate financial status (aOR = 1.51, 1.04–2.18, *p* = 0.03) being associated with higher willingness. Conclusions: Significant gaps exist in public awareness and willingness to vaccinate against HZ in Poland. Multifaceted strategies, including targeted media campaigns, enhanced physician engagement and improved access, are needed to increase vaccination rates.

## 1. Introduction

Herpes zoster is a viral disease resulting from the activation of latent neurotropic varicella zoster virus in the sensory ganglia after a previous primary varicella infection. Clinical manifestations of herpes zoster are observed in stages. The disease starts with an intense burning pain in the affected dermatome, accompanied by headache, malaise and photophobia. After one to three days, the characteristic unilateral rash appears in the form of multiple umbilicated vesicles, which produce ulcers and dry out in approximately 2 to even 4 weeks. The condition then proceeds to the chronic stage, manifesting in often very severe chronic pain, paresthesia and dysesthesias [1,2]. Despite the presence of circulating varicella zoster virus (VZV) antibodies [3], as cellular immunity diminishes, particularly with advancing age and due to medical conditions or medications, the level of specific memory T-cells decreases, causing the VZV to reactivate and produce symptoms [3,4].

As studies show, the main risk factors increasing the incidence of herpes zoster (HZ) are states of immunosuppression, such as in Human Immunodeficiency Virus (HIV)/Acquired Immune Deficiency Syndrome (AIDS), cancer or after organ or cell transplantation [5]. In addition, older age, history of physical trauma and female gender have been marked as contributing factors [6]. Many autoimmune disorders, such as systemic lupus erythematosus, irritable bowel disease, rheumatoid arthritis and psoriasis, often require immunosuppressive treatment [7], further increasing susceptibility to HZ. Additionally, various comorbidities, including coronavirus disease (COVID-19), cardiovascular diseases, chronic liver disease, renal disease, lung diseases, diabetes and depression [8], have been pointed to elevate the risk of HZ.

The incidence of herpes zoster (shingles) in Poland varies depending on the methodology and population studied. A review of regional data estimates the average incidence to be approximately 338.8 cases per 100,000 population annually, with significantly higher rates observed in individuals over 50 years of age, reaching approximately 614.3 per 100,000 in this group. The incidence increases with age, reflecting the waning immunity typically associated with aging. Hospitalization rates for severe complications remain relatively low, averaging around 5.42 cases per 100,000 population [9]. In a broader European context, Poland’s incidence aligns with the general pattern observed across the continent, where rates range from 2 to 4.6 cases per 1000 person-years [10].

Vaccination is the most effective measure to prevent HZ and its complications [11]. The adjuvant recombinant VZV vaccine has been shown to reduce the overall incidence of herpes zoster by 97% in older adults (>50 years old) in a large-scale, multi-center, double-blind, randomized study (ZOE-50) [12]. The concurrent study (ZOE-70) in participants 70 years or older reduced HZ incidence by 91.3% and showed vaccine efficacy against postherpetic neuralgia at 88.8% [13].

In Poland, the VZV vaccine was approved for use in 2023 and recommended for adults over 50 years. Since 1 January 2024, the VZV vaccine has been available across the country to patients 65 years and older from high-risk groups with 50% reimbursement of the cost from the National Health Fund [14]. It is also commercially available for adult patients. The Polish Society of Vaccinology expert group and several other organizations [8] recommend the vaccination to all patients 50 years and older and younger patients with selected comorbidities.

In recent years, most vaccine uptake promotion in higher-income countries focused on COVID-19 or influenza vaccination. The level of knowledge about the vaccine appears to vary greatly among different populations, from less than 10% [15] to around 40% [16]. The main sources of information indicated were media (press, TV, radio), friends, relatives and doctors. A study conducted in Italy examining vaccine uptake presented direct text messages as the most common reason to participate in vaccination [15]. This particular measure has been previously used in Poland during the first phase of the COVID-19 pandemic [17,18].

It has been observed that even for individuals who were reluctant to vaccinate against HZ, the physician’s recommendation could reverse as much as 70% of refusals [16], indicating a considerable potential for increasing vaccination rates.

To our knowledge, in Polish conditions, there have been no large-scale campaigns promoting the HZ vaccination. Most informative material is available online on governmental health agencies’ websites [16,19], which may decrease its accessibility to older adults.

Including the VZV vaccine in large-scale state-sponsored informational campaigns may contribute significantly to reducing the burden of the disease, especially as HZ is estimated to affect as much as 30% of the general population during their lifetime [4], of which 20% will suffer from postherpetic neuralgia. It is, therefore, essential to examine the level of knowledge about the HZ vaccine in the Polish population to provide effective promotional measures.

This study aimed to assess awareness of the shingles vaccine and identify factors associated with willingness to vaccinate against shingles in a nationwide sample of adults in Poland.

## 2. Materials and Methods

### 2.1. Study Design and Population

This cross-sectional survey was conducted in a nationwide sample of adults in Poland. Data were collected in September 2024 (between Friday 20th and Monday 23rd). The computer-assisted web interview (CAWI) method was used for data collection. The study questionnaire was available online on a dedicated research platform managed by the public opinion research company, the Ariadna Research Panel [20]. Each participant was invited to participate in the survey via e-mail and text message. The questionnaire could be filled out only once, and only completed questionnaires were collected.

Participants were selected from the dataset of 100,000 registered and verified users of the Ariadna Research Panel. Respondents were selected using non-probability quota sampling methods. The stratification model included sex, age, and place of residence. The study population reflected the demographic structure of the adult population of Poland, as published by the Statistics of Poland [21], which allowed the differentiation of groups of patients below and above the age the vaccination is recommended and reimbursed (50 and 65 years, respectively). If someone refused to participate in the survey, another participant with the same demographic characteristics was selected and invited to participate. The overall response rate was estimated at 23%. A total of 1137 adults in Poland completed the questionnaire.

The same methodology was used in previous nationwide studies on public attitudes toward vaccinations and vaccines [22,23].

Participation in this study was voluntary and anonymous. Each participant declared informed consent to participate in the study. The Ethics Committee at the Centre of Postgraduate Medical Education in Warsaw approved the study protocol (no. 29/2024 as of 15 May 2024).

### 2.2. Measures

The study questionnaire included seven questions on the shingles vaccine (Supplementary Material S1) prepared based on a literature review [24,25]. Moreover, media campaigns on vaccination against shingles performed after the vaccine launch in Poland were analyzed.

Awareness of the vaccination against shingles: “Have you ever heard about the vaccination against shingles?” (yes/no). Those who declared “yes” were asked to indicate all sources of knowledge on vaccination against shingles, with ten possible sources (multiple-choice questions). Moreover, all respondents were asked to indicate the shingles-vaccination-eligible populations and the symptoms of shingles.

Willingness to vaccinate against shingles: “Would you like to get vaccinated against shingles if you were in a group for which vaccination is recommended?” with a 5-point Likert scale. Respondents who declared definitely yes or rather yes were considered as those who declared willingness to vaccinate against shingles. Moreover, the following question was addressed: “Would you recommend vaccination against shingles to your relatives (parents, siblings, partner) if they were in the group for which vaccination is recommended?” with the same 5-point Likert scale. Respondents who declared definitely yes or rather yes were considered as those who declared willingness to recommend vaccination against shingles to their relatives.

Respondents were also asked questions regarding their socioeconomic status (i.e., gender, age, education, place of residence, marital status), which included a self-perceived assessment of their financial status (good, moderate or bad).

### 2.3. Data Analysis

Data were analyzed with IBM SPSS version 29 (Armonk, NY, USA). Categorical variables were presented with frequencies and proportions. The chi-squared test and cross-tabulations were used to compare categorical variables. Logistic Regression Models were used to identify factors associated with (1) public awareness of the shingles vaccine, (2) willingness to vaccinate against shingles, if eligible, and (3) willingness to recommend the shingles vaccine to relatives, if eligible. Ten different independent variables were identified and included independently in univariable analyses. Only variables statistically significant in bivariable analysis were included in multivariable regression models. The odds ratio (OR) and 95% confidence intervals (95%CI) were used to assess the strength of the associations. The statistical significance level was based on the criterion *p* < 0.05.

## 3. Results

The study population included 1137 adults aged 18–88; 55.6% were females (Table 1). Among the participants, 22.3% (*n* = 253) declared the presence of chronic diseases like diabetes, rheumatic diseases, post-transplantation status and cancer diagnosed by a doctor.

### 3.1. Public Awareness of VZV Vaccination

Awareness of vaccination against shingles was declared by 47% (*n* = 534) of participants (Table 2). Among those who claimed awareness of the shingles vaccine, TV was the major source of knowledge, indicated by 52.6% (*n* = 281) of respondents. Less than one-third of respondents indicated doctors as a source of knowledge on vaccination against shingles (Table 2). Adults aged 50 years and over were indicated as shingles-vaccination-eligible populations by 57.3% (*n* = 652) of participants, adults with chronic diseases by 60.5% (*n* = 688) of participants, and 71.2% (*n* = 809) of participants indicated patients with immune deficiencies as shingles-vaccination-eligible populations (Table 2). Among the respondents, 63.7% (37.6% rather yes and 26.1% definitely yes, *n* = 428 and *n* = 297, respectively) declared willingness to get vaccinated against shingles if they were in a group for which vaccination is recommended. Willingness to recommend vaccination against shingles to relatives (parents, siblings, partner) if they were in the group for which vaccination is recommended was declared by 49.8% of respondents (32.6% rather yes and 17.2% definitely yes, *n* = 371 and *n* = 196, respectively). The most common symptoms of shingles—like pain, numbness or itching of the skin, skin rash with blisters and sharp pain running along a specific part of the body—were indicated by over 60% of participants (Table 2).

### 3.2. Sociodemographic Differences in the Public Awareness of the VZV Vaccination

There were statistically significant differences in public awareness of the shingles vaccine based on marital status, having children, and the presence of chronic diseases (Table 3). When divided into three age groups (<50; 50–64 years and 65 years and over), there were no statistically significant differences (*p* = 0.07) in the percentage of respondents who declared awareness of the shingles vaccine.

In multivariable logistic regression (Table 3), only patients diagnosed with chronic disease had higher odds of declaring awareness of the shingles vaccine (aOR = 1.35, 1.02–1.80, *p* = 0.04).

### 3.3. Public Attitudes Towards VZV Vaccination

There were statistically significant differences in public attitudes towards willingness to vaccinate against shingles by having children and financial status (Table 4). When divided into three age groups (<50; 50–64 years and 65 years and over), there were no statistically significant differences (*p* = 0.3) in the percentage of respondents who declared willingness to vaccinate against shingles. In multivariable logistic regression (Table 4), participants who had not had children (aOR = 1.30, 1.01–1.69, *p* = 0.04) and moderate financial status (aOR = 1.51, 1.04–2.18, *p* = 0.03) were significantly associated with willingness to getting vaccinated against shingles.

### 3.4. Sociodemographic Differences in Recommending the VZV Vaccination

There were statistically significant differences in public attitudes towards willingness to recommend the shingles vaccine to relative (Table 5). When divided into three age groups (<50; 50–64 years and 65 years and over), there were statistically significant differences (*p* = 0.01) in the percentage of respondents who declared willingness to recommend the shingles vaccine to relatives (46.7%, 50.5% and 58.7%, respectively). In multivariable logistic regression (Table 5), participants aged 50 years and over (aOR = 1.35, 1.03–1.77, *p* = 0.03), having higher education (aOR = 1.60, 1.25–2.04, *p* < 0.001), good financial status (aOR = 1.68, 1.16–2.41, *p* = 0.01) and diagnosis of chronic diseases (aOR = 1.69, 1.26–2.28, *p* < 0.001) were significantly associated with willingness to recommend the shingles vaccine to relatives (Table 5).

## 4. Discussion

The findings of this study reveal considerable gaps in public awareness and willingness to vaccinate against shingles among the adult population in Poland. Despite introducing a reimbursement policy in 2024 and increasing the availability of the shingles vaccine, only 47% of respondents reported being aware of its existence. This is significantly lower than the 70% reported in some higher-income countries [24].

Television was identified as the primary source of information, underscoring the need for enhanced and diversified public health communication strategies. This aligns with findings from previous studies, which highlight the pivotal role of media campaigns in raising awareness of adult vaccination programs [25]. However, reliance on internet-based information disproportionately affects older populations, who may face digital literacy barriers, and suggests the need for supplementary communication approaches, such as community-based outreach and printed materials.

Willingness to vaccinate against shingles was expressed by 63.7% of participants if they were included in an eligible group, a proportion comparable to findings from other studies examining vaccine hesitancy [26]. Those with moderate financial status and those without children showed significantly greater willingness, likely indicating the influence of perceived affordability and reduced caregiving responsibilities. These findings resonate with research on barriers to vaccine uptake, where cost and competing responsibilities are common concerns [27]. Additionally, individuals with chronic diseases were more likely to recommend vaccination to relatives, highlighting patient advocacy’s role in promoting vaccine acceptance. Such peer-driven promotion may complement official public health campaigns, particularly among high-risk populations, as checked during the COVID-19 pandemic [28].

According to vaccine registration documents, in Poland, the VZV vaccine is available for adults over 50 years. Due to this fact, in this study, analyses were carried out for those aged under 50 years as well as 50 years and over. However, due to pharmacological and reimbursement reasons, the VZV vaccine is reimbursed 50% for those aged 65 and over. The amount of reimbursement is based on the decision of the Ministry of Health. Vaccine reimbursement may encourage older adults to vaccinate against shingles. However, the impact of the reimbursement may vary across different sociodemographic groups. Moreover, it is also unclear how physicians may approach the recommendation of a vaccine that is quite expensive, despite the 50% reimbursement.

A notable finding was the significant age-related disparity in vaccine awareness, with younger adults being less informed about the shingles vaccine than older participants. This contrasts with previous studies emphasizing younger populations as more receptive to health-related information campaigns, possibly indicating insufficiently targeting this group in Poland [29]. Furthermore, healthcare providers were an underutilized source of vaccine information, cited by only 30.5% of respondents. Previous research has demonstrated that physician recommendations are among the most influential factors in vaccine decision making, with a high potential to overcome hesitancy [30]. This emphasizes the need for healthcare professionals to actively promote vaccines during routine consultations and provide evidence-based advice tailored to individual patient needs.

The results also highlight gaps in the public’s understanding of shingles and its vaccine-eligible populations. While over 60% correctly identified common symptoms of shingles, misconceptions about vaccination eligibility persist. This calls for educational interventions that improve knowledge about the disease and clarify who can benefit most from vaccination. Comparable studies have shown that well-designed educational campaigns can significantly increase vaccine acceptance by addressing specific misconceptions and enhancing perceived benefits [26]. Additionally, the limited use of printed materials and the absence of large-scale campaigns targeting high-risk groups, such as older adults and individuals with chronic conditions, suggest an area for improvement in public health strategies.

The interplay of these factors highlights the complexity of improving vaccine uptake for shingles in Poland. Addressing these challenges will require a multifaceted approach, combining effective communication strategies with enhanced access to vaccines and proactive involvement of healthcare providers. Strengthening public health messaging through multiple channels and leveraging the trust placed in physicians can help bridge the gap in vaccine awareness and willingness [31], ultimately contributing to reducing shingles-related morbidity and its complications.

### 4.1. Practical Implications

The findings highlight the urgent need for public health interventions targeting key barriers to HZ vaccine uptake in Poland. These interventions should involve tailored communication strategies emphasizing the benefits of vaccination, particularly among older adults and those with chronic conditions. Increased collaboration between healthcare providers and public health initiatives is crucial to ensure optimal vaccine accessibility and utilization.

### 4.2. Strengths and Limitations

This study has several strengths that enhance the reliability and relevance of its findings. First, using a nationwide sample that reflects the demographic structure of the Polish adult population adds to the generalizability of the results. Second, applying the computer-assisted web interview (CAWI) method allowed for efficient data collection while minimizing interviewer bias. Furthermore, including a comprehensive set of sociodemographic and health-related variables enabled a detailed exploration of factors influencing public awareness and willingness to vaccinate against shingles. These aspects underscore the robustness of the study’s design and its potential to inform targeted public health interventions.

However, the study is not without limitations. The reliance on self-reported data may introduce recall and social desirability biases, potentially affecting the accuracy of responses regarding vaccine awareness and willingness. Additionally, while effective for ensuring demographic representation, the non-probability quota sampling approach and participants’ willingness to take part in the survey may limit the external validity to populations outside the study framework. Another limitation is the absence of longitudinal data, which precludes an assessment of causal relationships or changes in attitudes over time.

## 5. Conclusions

This study highlights significant gaps in public awareness and willingness to vaccinate against shingles in Poland despite the availability of a highly effective vaccine. While less than half of the respondents were aware of the vaccine, the willingness to vaccinate among eligible groups was moderate, influenced by factors such as financial status and health conditions. These findings emphasize the urgent need for tailored public health strategies to improve vaccine uptake, including large-scale awareness campaigns, integration of shingles vaccination into routine healthcare services and enhanced patient education.

Addressing these gaps is crucial to reducing the burden of shingles and its complications, particularly in aging and high-risk populations. By leveraging these insights, policy makers and healthcare providers can design effective interventions to increase vaccine coverage, ultimately contributing to preventing vaccine-preventable diseases and enhancing public health outcomes. Further research is needed to evaluate the long-term impacts of such strategies and refine approaches to improving adult vaccination rates globally.

## Figures and Tables

**Table 1:** Characteristics of the study population (*n* = 1137).

Variable	*n*	%
Gender		
female	632	55.6
male	505	44.4
Age (years)		
18–49	608	53.5
50+	529	46.5
Higher education		
yes	544	47.8
no	593	52.2
Married		
yes	589	51.8
no	548	48.2
Place of residence		
rural area	416	36.6
city < 20,000 inhabitants	151	13.3
city ≥ 20,000–99,999 inhabitants	225	19.8
city ≥ 100,000–499,999 inhabitants	195	17.2
city ≥ 500,000 inhabitants	150	13.2
Having children		
yes	741	65.2
no	396	34.8
Number of household members		
1 (living alone)	167	14.7
2	413	36.3
3 or more	557	49.0
Occupational activity		
active	704	61.9
passive	433	38.1
Financial status of the family		
good	533	46.9
moderate	430	37.8
bad	174	15.3
Chronic diseases (like diabetes, rheumatic diseases, post-transplantation status, cancer) diagnosed by a doctor		
yes	253	22.3
no	884	77.7

**Table 2:** Public awareness of the vaccination against shingles (*n* = 1137).

Variable	*n*	%
Have you ever heard about the vaccination against shingles?		
yes	534	47.0
no	603	53.0
Please indicate all sources from vaccination against shingles. (n = 534) (multiple-choice question)		
TV	281	52.6
radio	75	14.0
press	60	11.2
doctor	163	30.5
nurse	94	17.6
poster in a healthcare facility	115	21.5
internet advertising	74	13.9
social media	94	17.6
information on the web news portal	113	21.2
other sources	28	5.2
Please indicate the shingles-vaccination-eligible populations.		
correct answers		
adults aged 50 years and over	652	57.3
adults with chronic diseases	688	60.5
patients with immune deficiencies	809	71.2
incorrect answers		
all adults	508	44.7
adults aged 70 years and over	657	57.8
Would you like to get vaccinated against shingles if you were in a group for which vaccination is recommended?		
definitely no	111	9.8
rather no	145	12.8
rather yes	428	37.6
definitely yes	297	26.1
I don’t know	156	13.7
Would you recommend vaccination against shingles to your relatives (parents, siblings, partner) if they were in the group for which vaccination is recommended?		
definitely no	78	6.9
rather no	95	8.4
rather yes	371	32.6
definitely yes	196	17.2
I don’t know	353	31.0
I do not have any relatives who can be eligible population	44	3.9
What do you think are the symptoms of shingles?		
pain, numbness, or itching of the skin	729	64.1
skin rash with blisters	776	68.2
sharp pain running along a specific part of the body	698	61.4
headache	410	36.1
sore throat	198	17.4
feeling unwell	791	69.6
skin infection	565	49.7
low-grade fever or fever	732	64.4
lymphadenopathy	474	41.7

**Table 3:** Sociodemographic differences in public awareness of shingles vaccine (*n* = 1137).

	Awareness of Shingles Vaccine (Only “Yes” Answers Are Included in the Table)								
	Chi-Squared Test			Univariable Logistic Regression			Multivariable Logistic Regression		
Variable	n	%	*p*	OR	95%CI	*p*	aOR	95%CI	*p*
Gender									
female	302	47.8	0.5	1.08	0.85–1.36	0.5			
male	232	45.9		Ref.					
Age (years)									
18–49	272	44.7	0.1	0.83	0.65–1.04	0.1			
50+	262	49.5		Ref.					
Higher education									
Yes	248	45.6	0.4	0.90	0.71–1.14	0.4			
No	286	48.2		Ref.					
Married									
yes	299	50.8	**0.01**	1.37	1.09–1.74	**0.01**	1.29	0.99–1.70	0.06
no	235	42.9		Ref.			Ref.		
Place of residence									
rural area	183	44.0	0.4	0.95	0.65–1.38	0.8			
city < 20,000 inhabitants	69	45.7		1.02	0.65–1.60	0.9			
city ≥ 20,000–99,999 inhabitants	114	50.7		1.24	0.82–1.87	0.3			
city ≥ 100,000–499,999 inhabitants	100	51.3		1.27	0.83–1.95	0.3			
city ≥ 500,000 inhabitants	68	45.3		Ref.					
Having children									
yes	366	49.4	**0.03**	1.33	1.04–1.69	0.03	1.15	0.86–1.52	0.3
no	168	42.4		Ref.			Ref.		
Number of household members									
1 (living alone)	68	40.7	0.07	0.81	0.57–1.15	0.2			
2	210	50.8		1.22	0.94–1.57	0.1			
3 or more	256	46.0		Ref.					
Occupational activity									
active	316	44.9	0.07	0.80	0.63–1.02	0.07			
passive	218	50.3		Ref.					
**Financial status of the family**									
good	250	46.9	0.6	1.14	0.81–1.61	0.5			
moderate	208	48.4		1.21	0.85–1.72	0.3			
bad	76	43.7		Ref.					
Chronic diseases (like diabetes, rheumatic diseases, post-transplantation status, cancer) diagnosed by a doctor									
yes	134	53.0	**0.03**	1.36	1.03–1.80	**0.03**	1.35	1.02–1.80	**0.04**
no	400	45.2		Ref.			Ref.		

**Table 4:** Sociodemographic differences in willingness to vaccinate against shingles (*n* = 1137).

Willingness to Vaccinate Against Shingles (Only “Yes” Answers Are Included in the Table)									
	Chi-Squared Test			Univariable Logistic Regression			Multivariable Logistic Regression		
Variable	n	%	*p*	OR	95%CI	*p*	aOR	95%CI	*p*
Gender									
female	418	66.1	0.06	1.26	0.99–1.61	0.06			
male	307	60.8		Ref.					
Age (years)									
18–49	395	65.0	0.4	1.12	0.88–1.43	0.4			
50+	330	62.4		Ref.					
Higher education									
yes	342	62.9	0.5	0.93	0.73–1.18	0.5			
no	383	64.6		Ref.					
Married									
yes	361	61.3	**0.07**	0.80	0.63–1.02	**0.07**			
no	364	66.4		Ref.					
Place of residence									
rural area	270	64.9	0.2	1.04	0.71–1.54	0.8			
city < 20,000 inhabitants	98	64.9		1.04	0.65–1.67	0.8			
city ≥ 20,000–99,999 inhabitants	151	67.1		1.15	0.74–1.77	0.5			
city ≥ 100,000–499,999 inhabitants	110	56.4		0.73	0.47–1.13	0.2			
city ≥ 500,000 inhabitants	96	64.0		Ref.					
Having children									
yes	457	61.7	**0.04**	Ref.			Ref.		
no	268	67.7		1.30	1.01–1.68	**0.04**	1.30	1.01–1.69	**0.04**
Number of household members									
1 (living alone)	102	61.1	0.7	0.88	0.62–1.26	0.5			
2	266	64.4		1.01	0.78–1.32	0.9			
3 or more	357	64.1		Ref.					
Occupational activity									
active	445	63.2	0.6	0.94	0.73–1.21	0.6			
passive	280	64.7		Ref.					
Financial status of the family									
good	319	59.8	**0.04**	0.96	0.67–1.36	0.8	0.98	0.69–1.39	0.9
moderate	300	69.8		1.48	1.03–2.14	**0.04**	1.51	1.04–2.18	**0.03**
bad	106	60.9		Ref.			Ref.		
Chronic diseases (like diabetes, rheumatic diseases, post-transplantation status, cancer) diagnosed by a doctor									
yes	155	61.3	0.4	0.87	0.65–1.16	0.4			
no	570	64.5		Ref.					

**Table 5:** Sociodemographic differences in willingness to recommend shingles vaccine to relatives (*n* = 1137).

Willingness to Recommend Shingles Vaccine to Relatives (Only “Yes” Answers Are Included in the Table)									
	Chi-Squared Test			Univariable Logistic Regression			Multivariable Logistic Regression		
Variable	n	%	*p*	OR	95%CI	*p*	aOR	95%CI	*p*
Gender									
female	311	49.2	0.6	0.94	0.75–1.19	0.6			
male	256	50.7		Ref.					
Age (years)									
18–49	284	46.7	**0.02**	Ref.			Ref.		
50+	283	53.5		1.31	1.04–1.66	**0.02**	1.35	1.03–1.77	**0.03**
Higher education									
yes	307	56.4	**<0.001**	1.66	1.31–2.10	**<0.001**	1.60	1.25–2.04	**<0.001**
no	260	43.8		Ref.			Ref.		
Married									
yes	307	52.1	0.1	1.21	0.96–1.52	0.1			
no	260	47.4		Ref.					
Place of residence									
rural area	190	45.7	**0.02**	Ref.			Ref.		
city < 20,000 inhabitants	64	42.4		0.88	0.60–1.27	0.5	0.84	0.57–1.24	0.4
city ≥ 20,000–99,999 inhabitants	123	54.7		1.43	1.04–1.99	**0.03**	1.33	0.95–1.86	0.1
city ≥ 100,000–499,999 inhabitants	105	53.8		1.39	0.99–1.95	0.06	1.36	0.96–1.94	0.09
city ≥ 500,000 inhabitants	85	56.7		1.56	1.07–2.27	**0.02**	1.45	0.98–2.14	0.06
Having children									
yes	368	49.7	0.9	0.98	0.77–1.25	0.9			
no	199	50.3		Ref.					
Number of household members									
1 (living alone)	72	43.1	**0.01**	0.84	0.59–1.19	0.3	0.76	0.52–1.11	0.2
2	231	55.9		1.41	1.09–1.82	**0.01**	1.23	0.93–1.63	0.2
3 or more	264	47.4		Ref.			Ref.		
Occupational activity									
active	348	49.4	0.7	0.96	0.75–1.21	0.7			
passive	219	50.6		Ref.					
Financial status of the family									
good	298	55.9	**<0.001**	1.84	1.30–2.60	**<0.001**	1.68	1.16–2.41	**0.01**
moderate	198	46.0		1.24	0.87–1.77	0.2	1.12	0.77–1.62	0.6
bad	71	40.8		Ref.			Ref.		
Chronic diseases (like diabetes, rheumatic diseases, post-transplantation status, cancer) diagnosed by a doctor									
yes	151	59.7	**<0.001**	1.67	1.25–2.21	**<0.001**	1.69	1.26–2.28	**<0.001**
no	416	47.1		Ref.			Ref.		

## Data Availability

Data are available from the corresponding author on reasonable request.

## Supplementary Materials

The following supporting information can be downloaded at https://www.mdpi.com/article/10.3390/vaccines12121393/s1, Supplementary material S1: Study questionnaire.

## References

[1] Patil A., Goldust M., Wollina U. (2022). Herpes zoster: A Review of Clinical Manifestations and Management. Viruses.

[2] McCrary M.L., Severson J., Tyring S.K. (1999). Varicella zoster virus. J. Am. Acad. Dermatol..

[3] Gershon A.A., Gershon M.D. (2013). Pathogenesis and current approaches to control of varicella-zoster virus infections. Clin. Microbiol. Rev..

[4] Marra Y., Lalji F. (2022). Prevention of Herpes Zoster: A Focus on the Effectiveness and Safety of Herpes Zoster Vaccines. Viruses.

[5] Steinmann M., Lampe D., Grosser J., Schmidt J., Hohoff M.L., Fischer A., Greiner W. (2024). Risk factors for herpes zoster infections: A systematic review and meta-analysis unveiling common trends and heterogeneity patterns. Infection.

[6] Marra F., Parhar K., Huang B., Vadlamudi N. (2020). Risk Factors for Herpes Zoster Infection: A Meta-Analysis. Open Forum Infect. Dis..

[7] Gershon A.A., Breuer J., Cohen J.I., Cohrs R.J., Gershon M.D., Gilden D., Grose C., Hambleton S., Kennedy P.G., Oxman M.N. (2015). Varicella zoster virus infection. Nature reviews. Dis. Primers.

[8] Kuchar E., Rudnicka L., Kocot-Kępska M. (2023). Herpes zoster vaccination—Recommendations of the group of experts of the Polish Society of Vaccinology, the Polish Society of Family Medicine, the Polish Society of Dermatology, the Polish Association for the Study of Pain and the Polish Neurological Society. BÓL.

[9] Albrecht P., Patrzałek M., Goryński P. (2015). The burden of Herpes Zoster and its complications in Poland in according to the age. Przegl Epidemiol..

[10] Pinchinat S., Cebrián-Cuenca A.M., Bricout H., Johnson R.W. (2013). Similar herpes zoster incidence across Europe: Results from a systematic literature review. BMC Infect Dis..

[11] Saguil A., Kane S., Mercado M., Lauters R. (2017). Herpes Zoster and Postherpetic Neuralgia: Prevention and Management. Am. Fam. Physician.

[12] Lal H., Cunningham A.L., Godeaux O., Chlibek R., Diez-Domingo J., Hwang S.J., Levin M.J., McElhaney J.E., Poder A., Puig-Barberà J. (2015). Efficacy of an adjuvanted herpes zoster subunit vaccine in older adults. N. Engl. J. Med..

[13] Cunningham A.L., Lal H., Kovac M., Chlibek R., Hwang S.J., Díez-Domingo J., Godeaux O., Levin M.J., McElhaney J.E., Puig-Barberà J. (2016). Efficacy of the Herpes Zoster Subunit Vaccine in Adults 70 Years of Age or Older. N. Engl. J. Med..

[14] National Institute of Public Health—National Research Institute Szczepionka Przeciw Półpaścowi Objęta 50% Refundacją. https://szczepienia.pzh.gov.pl/szczepionka-przeciw-polpascowi-objeta-50-refundacja/.

[15] Valente N., Lupi S., Stefanati A., Cova M., Sulcaj N., Piccinni L., Gabutti G., GPs Study Group (2016). Evaluation of the acceptability of a vaccine against herpes zoster in the over 50 years old: An Italian observational study. BMJ Open.

[16] Yang T.U., Cheong H.J., Song J.Y., Noh J.Y., Kim W.J. (2015). Survey on public awareness, attitudes, and barriers for herpes zoster vaccination in South Korea. Hum. Vaccines Immunother..

[17] Communication form the Polish Ministry of Health of 21.11.2021. https://www.gov.pl/web/zdrowie/sms-z-ministerstwa-zdrowia-do-osob-50plus.

[18] Communication form the Polish Ministry of Health of 11.01.2022. https://www.gov.pl/web/zdrowie/nowe-sms-y-z-ministerstwa-zdrowia.

[19] National Institute of Public Health—National Research Institute Kalendarz Szczepień Osób Starszych. https://szczepienia.pzh.gov.pl/kalendarz-szczepien-osob-starszych-2/.

[20] Nationwide Research Panel Ariadna. https://panelariadna.com/.

[21] Statistics of Poland Demographic Yearbook of Poland 2022. https://stat.gov.pl/en/topics/statistical-yearbooks/statistical-yearbooks/demographic-yearbook-of-poland-2022,3,16.html.

[22] Kamińska A., Jankowski M., Rejdak M.B., Ostrowski J., Rejdak R., Pinkas J. (2023). An Online Questionnaire-Based Survey of 1076 Individuals in Poland to Identify the Prevalence of Ophthalmic Symptoms in Autumn. Med Sci Monit..

[23] Jankowski M., Grudziąż-Sękowska J., Wrześniewska-Wal I., Tyszko P., Sękowski K., Ostrowski J., Gujski M., Pinkas J. (2023). National HPV Vaccination Program in Poland-Public Awareness, Sources of Knowledge, and Willingness to Vaccinate Children against HPV. Vaccines.

[24] Wang Q., Yang L., Li L., Liu C., Jin H., Lin L. (2023). Willingness to Vaccinate Against Herpes Zoster and Its Associated Factors Across WHO Regions: Global Systematic Review and Meta-Analysis. JMIR Public Health Surveill..

[25] Dooling K.L., Guo A., Patel M., Lee G.M., Moore K., Belongia E.A., Harpaz R. (2018). Recommendations of the Advisory Committee on Immunization Practices for Use of Herpes Zoster Vaccines. MMWR Recomm. Rep..

[26] Schmid P., Rauber D., Betsch C., Lidolt G., Denker M.L. (2017). Barriers of influenza vaccination intention and behavior: A systematic review of influenza vaccine hesitancy, 2005–2016. PLoS ONE.

[27] George S., Regan J., Awan A., O’Connor M., Foster A., Raymond K., Gorfinkel I., McNeil S.A. (2024). Attitudes, barriers, and facilitators to adherent completion of the recombinant zoster vaccine regimen in Canada: Qualitative interviews with healthcare providers and patients. Hum. Vaccines Immunother..

[28] Marquez C., Kerkhoff A.D., Naso J., Contreras M.G., Castellanos Diaz E., Rojas S., Peng J., Rubio L., Jones D., Jacobo J. (2021). A multi-component, community-based strategy to facilitate COVID-19 vaccine uptake among Latinx populations: From theory to practice. PLoS ONE.

[29] Kumar D., Chandra R., Mathur M., Samdariya S., Kapoor N. (2016). Vaccine hesitancy: Understanding better to address better. Isr. J. Health Policy Res..

[30] Czajka H., Czajka S., Biłas P., Pałka P., Jędrusik S., Czapkiewicz A. (2020). Who or What Influences the Individuals’ Decision-Making Process Regarding Vaccinations?. Int. J. Environ. Res. Public Health..

[31] Stasiuk K., Polak M., Dolinski D., Maciuszek J. (2021). The Credibility of Health Information Sources as Predictors of Attitudes toward Vaccination-The Results from a Longitudinal Study in Poland. Vaccines.

