# Supplementary material for: Public Awareness and Willingness to Vaccinate Against Herpes Zoster: A Nationwide Cross-Sectional Study in Poland
Source: Vaccines (Basel). 2024 Dec 11;12(12):1393. doi: 10.3390/vaccines12121393 (PMC11680232; doi:10.3390/vaccines12121393)
Supplement: Supplementary file 1 [file vaccines-12-01393-s001.zip › vaccines-3351610-supplementary.pdf]

**ANNEX no. 1**

## Questionnaire

**Information for participants**

Chickenpox and shingles virus (VZV - Varicella Zoster Virus). Primary infection with this virus causes chickenpox. After recovery from chickenpox, the VZV virus remains in the body in the dorsal ganglia in a dormant, inactive form, which is referred to as viral latency. It may become active again in the future, for example as a result of age-related immune decline. Reactivation of VZV infection clinically manifests itself as shingles. It is not possible to become infected with herpes zoster, but people who are not immunised (unvaccinated or who have not had the disease) can become infected with chickenpox from a sick person.

**[Q1] Have you ever heard about the vaccination against shingles?**

- yes
- no

**[Q2] Please indicate all sources from vaccination against shingles?**

To be asked only if the answer to Q1=" yes" [multiple-choice question]
- TV
- radio
- press
- doctor
- nurse
- poster in a healthcare facility
- Internet advertising
- social media
- information on the web news portal
- other sources

**[Q3] Please indicate the shingles-vaccination-eligible populations?**

[possible answers for each group: "yes" or "no" or "I don't know"]
- all adults
- adults aged 50 years and over
- adults aged 70 years and over
- adults with chronic diseases
- patients with immune deficiencies

**[Q4] The currently available vaccination against VZV, the virus that causes shingles, is recommended for people over 50 years of age and those with chronic diseases (e.g. diabetes, rheumatism, organ transplant, oncology treatment) or immunocompromised.**

**Would you like to get vaccinated against shingles if you were in a group for which vaccination is recommended?**
- definitely no
- rather no
- I do not know
- rather yes
- definitely yes

**[Q5] Would you recommend vaccination against shingles to your relatives (parents, siblings, partner) if they were in the group for which vaccination is recommended?**

- definitely no
- rather no
- I do not know
- rather yes
- definitely yes
- I do not have any relatives who can be eligible population

**[Q6] What do you think are the symptoms of shingles?**

[possible answers for each group: "yes" or "no" or "I don't know"]

- pain, numbness, or itching of the skin
- skin rash with blisters
- sharp pain running along a specific part of the body
- headache
- sore throat
- feeling unwell
- skin infection
- low-grade fever or fever
- Lymphadenopathy

**[Q7] Have you been diagnosed with a chronic disease (e.g. diabetes, rheumatic diseases, post-transplantation status, cancer) by a doctor?**

- yes
- no

**Questions regarding the socio-economic status of the study's participants:**

**Q1. Gender**

- female
- male

**Q2. Age**

- 18-24 years
- 25-34 years
- 35-44 years
- 45-54 years
- 55+ years

**Q3. Education**

- elementary
- vocational
- secondary
- higher (university)

**Q4. Marital status**

- married
- single

**Q5. Place residence.**

- rural area
- city < 20,000 inhabitants
- city ≥ 20,000 - 99,999 inhabitants
- city ≥ 100,000 - 499,999 inhabitants
- city ≥ 500,000 inhabitants

**Q6. Children (living in the same household)**

- yes
- no

**Q7. Number of household members**

- 1 (living alone)
- 2
- 3 or more

**Q8. Occupational status**

- active
- passive

**Q9. Financial status of the family**

- good
- moderate
- bad
